# A robust and reliable methodology to perform GECI-based multi-time point neuronal calcium imaging within mixed cultures of human iPSC-derived cortical neurons

**DOI:** 10.3389/fnins.2023.1247397

**Published:** 2023-09-25

**Authors:** Niraj Patel, Vincent Ouellet, François Paquet-Mercier, Nizar Chetoui, Erik Bélanger, Marie-Eve Paquet, Antoine G. Godin, Pierre Marquet

**Affiliations:** ^1^Department of Psychiatry and Neuroscience, Laval University, Quebec, QC, Canada; ^2^CERVO Brain Research Centre, Laval University, Quebec, QC, Canada; ^3^Department of Biochemistry, Microbiology and Bioinformatics, Laval University, Quebec, QC, Canada; ^4^Centre for Optics, Photonics and Lasers (COPL), Laval University, Quebec, QC, Canada

**Keywords:** human iPSCs (induced pluripotent stem cells), neurodifferentiation, GECI (genetically encoded Ca^2+^ indicator), human synapsin1 (hSyn) promoter, CAG promoter, calcium imaging, disease modeling, neuronal network

## Abstract

**Introduction:**

Human induced pluripotent stem cells (iPSCs), with their ability to generate human neural cells (astrocytes and neurons) from patients, hold great promise for understanding the pathophysiology of major neuropsychiatric diseases such as schizophrenia and bipolar disorders, which includes alterations in cerebral development. Indeed, the *in vitro* neurodifferentiation of iPSCs, while recapitulating certain major stages of neurodevelopment *in vivo*, makes it possible to obtain networks of living human neurons. The culture model presented is particularly attractive within this framework since it involves iPSC-derived neural cells, which more specifically differentiate into cortical neurons of diverse types (in particular glutamatergic and GABAergic) and astrocytes. However, these *in vitro* neuronal networks, which may be heterogeneous in their degree of differentiation, remain challenging to bring to an appropriate level of maturation. It is therefore necessary to develop tools capable of analyzing a large number of cells to assess this maturation process. Calcium (Ca^2+^) imaging, which has been extensively developed, undoubtedly offers an incredibly good approach, particularly in its versions using genetically encoded calcium indicators. However, in the context of these iPSC-derived neural cell cultures, there is a lack of studies that propose Ca^2+^ imaging methods that can finely characterize the evolution of neuronal maturation during the neurodifferentiation process.

**Methods:**

In this study, we propose a robust and reliable method for specifically measuring neuronal activity at two different time points of the neurodifferentiation process in such human neural cultures. To this end, we have developed a specific Ca^2+^ signal analysis procedure and tested a series of different AAV serotypes to obtain expression levels of GCaMP6f under the control of the neuron-specific human synapsin1 (hSyn) promoter.

**Results:**

The retro serotype has been found to be the most efficient in driving the expression of the GCaMP6f and is compatible with multi-time point neuronal Ca^2+^ imaging in our human iPSC-derived neural cultures. An AAV2/retro carrying GCaMP6f under the hSyn promoter (AAV2/retro-hSyn-GCaMP6f) is an efficient vector that we have identified. To establish the method, calcium measurements were carried out at two time points in the neurodifferentiation process with both hSyn and CAG promoters, the latter being known to provide high transient gene expression across various cell types.

**Discussion:**

Our results stress that this methodology involving AAV2/retro-hSyn-GCaMP6f is suitable for specifically measuring neuronal calcium activities over multiple time points and is compatible with the neurodifferentiation process in our mixed human neural cultures.

## Introduction

The development of human induced pluripotent stem cells (iPSCs) has opened a new spectrum in the field of biomedical research, which allows the development of *in vitro* cellular disease models directly from patients to study the pathophysiology of diseases. Indeed, iPSCs are derived from somatic cells, which can be easily collected from patients (*e.g.,* fibroblasts from skin biopsy and urothelial cells from urine samples) and then transformed into selected differentiated cell types by adding the necessary growth/differentiation proteins and co-factors to the cell culture medium (Takahashi et al., 2007; Sharma, 2016). Specifically, iPSC-derived neuronal cells provide a unique model for identifying important elements in the pathophysiology of certain neuropsychiatric diseases, which can lead to the detection of biomarkers (Zhu et al., 2023), particularly in the context of personalized medicine since each cell line is derived from a specific patient. However, these iPSC-derived neural cultures remain delicate to bring to maturation, especially when neurodifferentiation is performed to obtain a set of different cortical neurons (Kim and Schnitzer, 2022), as presented in this article. Although the gold standard for assessing neuronal functional maturation is undoubtedly electrophysiological methods, given that these iPSC-derived neuronal cultures can be quite heterogeneous in terms of maturation, techniques enabling the evaluation of many neurons within a culture are highly relevant.

The calcium (Ca^2+^) imaging framework is particularly suitable for evaluating the maturation of these iPSC-derived neuronal cultures as it is widely used to assess neuronal activity in hundreds of neurons not only in culture but also in behaving mammals (Kim and Schnitzer, 2022). It is well beyond the scope of this article to present an exhaustive overview of Ca^2+^ cell imaging, which is so vast in terms of methodologies and applications, but in brief, we can mention that there are two main categories: one using chemical-based Ca^2+^ indicators and the other using genetically encoded calcium indicators (GECIs). In both cases, the fluorescence signals can be modulated in response to any changes in the intracellular Ca^2+^ levels.

Chemical-based Ca^2+^ indicators, such as membrane-permeant dye esters probes (Kantevari et al., 2011), whose Fluo4AM and Cal529AM are particularly well suited to investigate Ca^2+^ (Cobbold and Rink, 1987), require an incubation prior to imaging to allow cell loading. Once inside the cell, the AM ester groups are cleaved by esterase, resulting in a negatively charged fluorescent dye that stays inside the cells. In addition, due to uneven dye loading, membrane permeability alterations, photobleaching, and extrusion of cytosol into extracellular space, the imaging time with these chemical-based Ca^2+^ indicators can be limited between 1 and 4 h, depending on the dye used (Paredes et al., 2008; Tada et al., 2014). However, chemical-based Ca^2+^ indicators offer a wide range of Ca^2+^ affinities that are readily available to researchers (Paredes et al., 2008). Additionally, users may also have the option to use multiple indicators on the same experiment at different time points. Selecting the most suitable Ca^2+^ indicator depends on the specific experimental design and conditions.

Most GECI-based neuronal Ca^2+^ studies utilize the GCaMP family of indicators. GCaMPs consist of a Ca^2+^-binding calmodulin domain fused to a GFP. The GCaMP, incorporated into the genome, can be targeted to distinct populations of cells using cell-specific promoters and can help study multi-time point Ca^2+^ imaging of specific cells (Mao et al., 2008). Within the GCaMPs family, initially developed in 2001 (Nakai et al., 2001), there are several versions with distinct kinetics, dissociation constants of Ca^2+^ binding, and fluorescence levels when bound or unbound to Ca^2+^ (Dana et al., 2019). These parameters shape fluorescence waveforms in response to one or more neural events. Although the recent development of the 8th generation jGCaMP8 (Zhang et al., 2023) is particularly well suited to monitor Ca^2+^ changes from single events with greater precision compared to earlier generations, the 6th generation (GCaMP6), which includes three variants, GCaMP6s, 6 m, and 6f (for slow, medium, and fast Ca^2+^ signal response kinetics), has been extensively used to measure neuronal Ca^2+^ activity. In this well-established 6th generation, GCaMP6f was found to be particularly efficient in terms of its affinity for Ca^2+^, kinetics, and dynamic range (Chen et al., 2013; Badura et al., 2014).

However, in the field of human neuronal cultures derived from iPSCs, the use of GECI using a neuron-specific promoter, such as human synapsin1 (hSyn), to measure their activity notably through Ca^2+^ monitoring, has not yet been extensively studied. In recent years, a few studies have been conducted using the hSyn promoter on iPSC-based neuronal cultures, with a particular focus on studying specific neuron types or specific genetic mutations. However, most of these studies have primarily concentrated on molecular investigations, including protein and immunoassay analyses. For instance, a novel molecular tool was developed by combining the hSyn promoter and hVGAT-mCherry (human vesicular GABA transporter) with lentivirus (LV) to study GABAergic neuron development derived from iPSCs (DeRosa et al., 2015). Moreover, an LV-based hSyn promoter was employed to examine the neuronal development process in CDKL5 gene deficiency disorders such as seizure, which led to the identification of altered neuronal development in these pathological conditions (Negraes et al., 2021). Another notable molecular study conducted by Kassan *et al*. explored the regulation of the Caveolin-1 gene in schizophrenia using the SynCav1 promoter through an AAV9 viral construct (Kassan et al., 2017). In addition, serotonergic neurons generated from fibroblasts were transduced with hSynapsin;DsRed reporter lentiviral particles to characterize their functionality in order to develop a therapeutically relevant disease model (Vadodaria et al., 2016).

The aim of this study was to develop an efficient methodology to perform multi-time point Ca^2+^ imaging using GECI in human iPSC-derived neurons. Specifically, we consider cultures of human iPSC-cortical neurons containing several types of neurons as well as astrocytes that help their maturation, a culture model particularly useful for studying neuropsychiatric diseases (Hong et al., 2023). Concretely, we chose to use the GCaMP6f variant, given its well-established reliability and efficacy, with the neuron-specific hSyn promoter to specifically measure neuronal Ca^2+^ within such mixed cultures. hSyn promoter results were also compared with those of the CAG promoter, which is known for its ability to sustain stable gene expression, maintain high recombinant protein expression for extended periods, and provide high transient gene expression across various cell types (Foecking and Hofstetter, 1986; Damdindorj et al., 2012, 2014).

Furthermore, to obtain an efficient and robust methodology, (1) a set of different constructs based on AAV2 vectors has been tested to ensure reliable viral infection efficiency and (2) a Ca^2+^ signal processing procedure for highly efficient detection of spontaneous Ca^2+^ events over multiple time points has been developed. Building on these developments, Ca^2+^ imaging to evaluate the neuronal network activity of such human iPSC-cortical neurons, performed at two time points with both constructs AAV2/retro_hSyn_GCaMP6f and AAV2/retro_CAG_GCaMP6f, is presented.

## Materials and methods

### Participants and iPSCs derivation

The iPSC lines used in this study were generated and provided by the iPSC Québec platform, LOEX, Québec, Canada. These iPSCs were derived from urothelial cells isolated from urine samples of three middle-aged men (Table 1). The derived iPSC clones were characterized and quantified to evaluate the iPSC quality using various techniques, such as immunoassays, RT-PCR to assess the expression of iPSC markers (Oct4, Nanog, Dnm3tb, hTERT, and Rex1), analysis of 15 short tandem repeats for genotyping, amelogenin analysis for sex determination, and G-banding for karyotyping. Subsequently, a well-validated iPSC clone from each line was transported to our laboratory for further study. Then, iPSCs were thawed, expanded, and maintained using hESC-qualified matrigel-coated plates (356277, Corning) with mTeSR™1 iPSC medium (85850, STEMCELL Technologies) as per the commercially available protocol from STEMCELL Technologies. The iPSCs were passaged when they reached approximately 70%–80% confluency using 0.5 mM EDTA (Ethylenediaminetetraacetic acid, S311-500, Fisher Scientific) prepared in PBS (10010023, Thermo Fisher Scientific).

**Table 1:** Information of human iPSCs and healthy control donors.

No.	iPSCs line ID	Age	Gender	Group
1	9062	44	Male	Healthy control
2	9001	34	Male	Healthy control
3	5439	44	Male	Healthy control

### Differentiation of human iPSCs to neuronal networks

#### Generation of embryoid bodies (EBs) from iPSCs

EBs that represent three embryonic germ layers were generated from the iPSC lines passage no. 14–18. The neural induction medium (05893, STEMCELL Technologies) and AggreWell™ 800 microwell culture plates (34815, STEMCELL Technologies) were used to form EBs by following step by step the commercially available protocol “STEMdiff™ neural system to generation and culture of neural progenitor cells” of STEMCELL Technologies. Practically, AggreWell™ plates allow for controlling the EBs’ size and having a uniform population. Once EBs were generated, they were transferred into a six-well plate to form rosettes. After 4–5 days, the rosettes were isolated using STEMdiff™ neural rosette selection reagent (05832, STEMCELL Technologies) and transferred into six-well plates to generate neuronal progenitor cells (NPCs). Once NPCs got around 80%–90% confluence, they were passaged and collected using accutase (07922, STEMCELL Technologies) and cryopreserved with STEMdiff™ neural progenitor freezing medium (05838, STEMCELL Technologies) in liquid nitrogen for further uses.

#### Differentiation of NPCs into neural cells

In contrast to the primary steps to generate NPCs from iPSCs, which are quite like any other protocol, various methods have been developed to differentiate NPCs into mixed cortical neural cultures. These distinct methods lead to slightly different results from each other (Galiakberova and Dashinimaev, 2020). The neuronal differentiation, morphology, and network activity of neurons can depend on the specific cultivation conditions, and this can also depend on the iPSC line used, the presence of growth factors in the culture medium, and the type of substrate (Zhang et al., 2013; Schörnig et al., 2021).

To achieve neural culture, differentiation and maintenance processes followed a commercially available, well-tested, effective, and highly validated protocol to generate cortical neurons based on BrainPhys™ basal serum-free supplements. This protocol results in improved spontaneous and evoked action potentials and network spontaneous Ca^2+^ activity (Bardy et al., 2015) and fastens the neurodifferentiation and maturation processes (Jackson et al., 2018). Concretely, the NPCs were thawed and maintained in STEMdiff™ neural progenitor medium (05833, STEMCELL Technologies). When NPCs reached up to 80%–90% confluency, they were passaged using accutase. Finally, NPC passages 4–5 proceeded to differentiate into neural cells. For neurodifferentiation, sterile 12-mm glass coverslips (CSs) were put in an individual well of a 24-well plate. Then, CSs were incubated at room temperature with 15 μg/mL poly-L-ornithine (PLO, A-00-4C, Millipore Sigma) for 3 h. The PLO solution was then removed, and the wells were washed 2–3 times with PBS. After that, 10 μg/mL of laminin (L2020-1MG, Sigma-Aldrich) solution (dissolved in PBS) was added into all wells, and culture plates were incubated for 2–3 h at room temperature. Then, laminin was removed and washed with PBS twice, then kept aside until NPCs were ready to seed. NPCs were counted and seeded at 5 k cells/cm^2^ per well, and 1 mL of NPC medium was added to each well. After 24 h, 50% of the NPCs medium was replaced with the BrainPhys™ neuronal medium (05790, STEMCELL Technologies) supplemented with NeuroCult™ SM1 neuronal supplement (05711, STEMCELL Technologies), N2 supplement-A (07152, STEMCELL Technologies), BDNF (20 ng/mL, 78005, STEMCELL Technologies), GDNF (20 ng/mL, 78058, STEMCELL Technologies), dibutyryl-cAMP (1 mM, 73882, STEMCELL Technologies), and L-ascorbic acid (200 nM, 4055/50, Tocris). After that, every 2–3 days, 50% of the neural culture medium is replaced by BrainPhys™ with supplements. Additionally, 1 μg/mL of laminin was added weekly to the neuronal culture medium. All cell cultures were incubated at 37°C with 5% CO_2_ and maintained for up to 6 weeks to study the neuronal network activity. In a pilot study of neural culture immunoassay, it was observed that the neural culture predominantly consisted of excitatory glutamatergic neurons (Vglut1 marker) and a lesser population of inhibitory GABAergic neurons (GAD65/67 marker) in both time points of the neurodifferentiation process, *i.e.*, on week 3 and week 6 (data not shown).

### Viral infection and expression of GECI variants in iPSC-derived neural culture

All viral vectors were prepared and packaged for infection at the viral vector core facility of the Canadian neurophotonics platform, CERVO Brain Research Centre, Québec, Canada (RRID:SCR_016477). Viral infection experiments were conducted on iPSC-neural cultures using various serotype combinations as detailed in Supplementary Table S1. The primary objective was to optimize viral infection efficiency and assess expression levels. From systematic exploration, it emerged that the most efficient constructs for Ca^2+^ imaging experiments were AAV2/retro_hSyn_GCaMP6f, which is neuron-specific, and AAV2/retro_CAG_GCaMP6f, which exhibits global expression. Furthermore, three different concentrations of genome copies per milliliter (GC/mL) were also examined: 0.5 × 10^10^ GC/mL, 1 × 10^10^ GC/mL, and 2 × 10^10^ GC/mL. Insufficient fluorescence expression was observed at a concentration of 0.5 × 10^10^ GC/mL. However, no difference in terms of fluorescence expression or activity level was observed between the concentrations of 1 × 10^10^ GC/mL and 2 × 10^10^ GC/mL (data not shown). Consequently, a concentration of 1 × 10^10^ GC/mL was used for the experiments.

The GECI viral infection of neural cultures was performed 10 days before the live-cell Ca^2+^ imaging experiments. For viral infection, the complete 1 mL of neuronal medium was removed from wells, and 500 μL of fresh neuronal medium with 1 × 10^10^ GC/mL was added per well. Following a GECI viral infection incubation period of 4–5 h, an additional 500 μL of neuronal medium was added to maintain a total volume of 1 mL per well. After 48 h, complete medium changes were carried out, followed by 50% medium changes every 2–3 days until the day of experiments.

### Reagents preparation for Ca^2+^ imaging

The neural cultures were perfused with an artificial CSF (ACSF) containing the following (mM): NaCl 135 (S6191, Sigma), KCl 5 (60128, Sigma), CaCl_2_ 2 (21097, Sigma), MgCl_2_^.^6H_2_O 2 (M33-500, Fisher Scientific), NaH_2_PO_4_ 2 (S5761, Sigma), HEPES 20 (BP310-100, Fisher Scientific), and Dextrose 10 (D16-1, Fisher Scientific). The pH was maintained between 7.2 and 7.4, and the osmolarity was 310–320 mOsm/L. The ACSF solution, prepared a day before each experiment and stored at 4°C, was utilized within 24 h. Moreover, L-glutamic acid (30 μM, 56860, Tocris) and TTX (1 μM, Cedarlane Labs) were prepared for additional experiments.

### Live cell Ca^2+^ imaging

For the Ca^2+^ imaging experiments, two batches of neurodifferentiation were conducted for each of the three iPSC lines, resulting in a total of six batches for every time point. For each time point of the three iPSC lines, 3–4 CSs from the two batches were examined. In preparation for live Ca^2+^ imaging experiments, the neural culture medium was replaced with ACSF buffer and acclimated for 30 min in an incubator before imaging, ensuring optimal conditions for the imaging process. Then, CSs were transferred into a custom-designed perfusion chamber (Bélanger et al., 2019, 2022). During the Ca^2+^ imaging, the perfusion chamber and ACSF buffer temperature were maintained at 37°C while the buffer flow rate was 1 mL/min (Lévesque et al., 2018). The cytosolic Ca^2+^ signals were recorded using a customized multimodal epifluorescence microscope (DHM-T1003, Lyncée Tec) (Bélanger et al., 2018). The light source used for fluorescence excitation is a 120-W lamp (X-Cite 120PC Q, Excelitas) with a FITC filter cube (FITC-5050A-000, Semrock). The epifluorescence signal is detected with a CCD camera (INFINITY3S-1UR, Lumenera) with a resolution of 1,329 × 1,080 pixels. Images were acquired at 10 Hz for 5 min with a magnification of 20× and a 0.70-numerical aperture (NA). A short terminal application of L-glut pulses for 30 s was applied after 180 s of baseline recording to distinguish neurons in a neural culture (Pickering et al., 2008). Throughout the experiment, three fields of view were recorded for each CS, and approximately 30–40 cells were imaged per field of view to assess Ca^2+^ activity. The Ca^2+^ data were acquired and stored in a tiff format for offline analysis.

### Immunocytochemistry

The cells were fixed for 15 min at room temperature using a 4% paraformaldehyde solution (J19943-K2, Thermo Fisher Scientific). Then, they were washed and permeabilized for 5 min with 0.1% Triton X-100 (AC215682500, Fisher Scientific) in PBS. The cells were then incubated with a mixture of 10% normal goat serum (P131873, Fisher Scientific) and 1% BSA (SH3057402, Fisher Scientific) in PBS to block the nonspecific bindings for 60 min. After that, the cells were incubated overnight at 4°C with the following primary antibodies: NeuN (1:50, mouse monoclonal, MAB377M, Fisher Scientific), GFAP (1:1,000, rabbit polyclonal, 60128, STEMCELL Technologies), MAP2 (1:1,000, chicken polyclonal, ab92434, Abcam), and GFP tag (1:2,000, chicken polyclonal, ab13970, Abcam), which were prepared in blocking solution. The following day, the cells were washed with PBS and incubated with secondary antibodies, Alexa Fluor 488 (1:1,000, ab150077, Abcam), or Alexa Fluor 568 (1:1,000, ab175473, Abcam), based on the primary antibody host species, for 1 h. Then, the cells were washed with PBS and incubated with DAPI solution (4′,6-diamidino-2-phenylindole, dihydrochloride, 1:10,000, D1306, Fisher Scientific) for 3 min to detect all nuclei. Subsequently, the cells were washed with PBS and then immersed in dH_2_O until mounted. The CSs were mounted with Fluoromount-G (0100-01, Southern Biotech), and all glass slides were placed in a dark chamber for 24 h to facilitate drying.

The visualization of the targeted molecule was performed with a confocal microscope (LSM 700, Zeiss). From each iPSC’s lines, 1–2 CSs resulting in 3–4 fields of view were randomly captured using a Z-stack image function with an objective of 20× and a NA of 0.50. The maximum intensity projection function was used to get the final image, and all cell counting was performed in the ImageJ software (v1.5.3 t, NIH).

### Statistics

Statistical analysis was performed with GraphPad Prism version 9.4. A T-test (two-tailed, unpaired) was conducted to compare all iPSC-derived neural cell immunoassays and neuronal network activity data between the hSyn and CAG promoter groups.

## Results

### Efficiency assessment of different viral constructs

**Figure 1:**
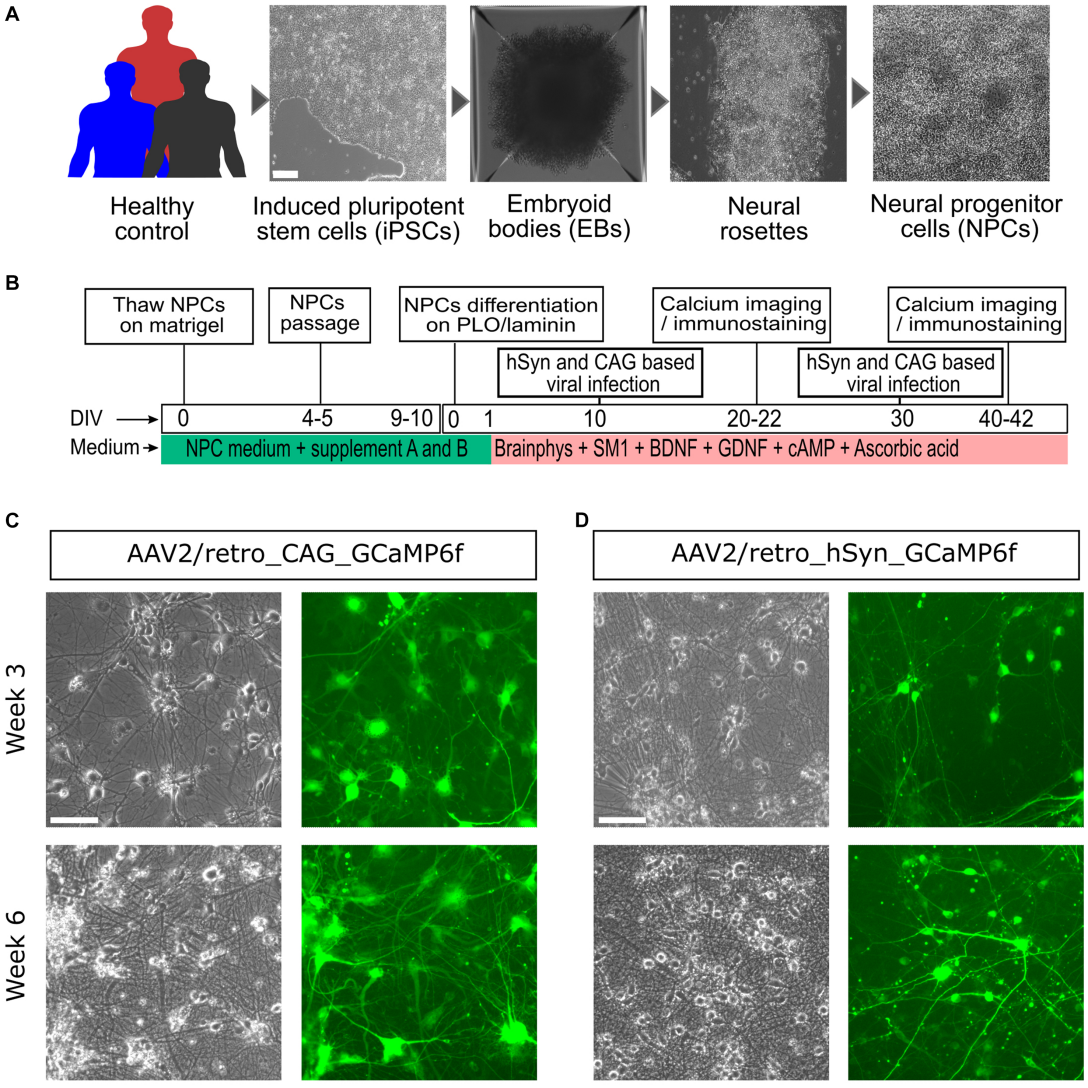
Generation, differentiation, and viral infection process of iPSC-derived neural culture. **(A)** NPCs were generated from human iPSCs derived from urothelial cells using a Sendai virus vector packed with the Yamanaka factors (Oct3/4, Sox2, Klf4, and c-Myc) (scale bar = 100 μm). **(B)** The neural culture generated from the NPCs and medium supplements was used for neural cell differentiation, maturation, and maintenance *in vitro*. At DIV10, the neural culture was then infected with AAV2/retro-expressing GCaMP6f (under the hSyn or CAG promoter), and live Ca^2+^ cell imaging was achieved on weeks 3 and 6. **(C)** Representative images taken on day 10 after viral infection, showing a phase contrast image (left) and a fluorescence image (right) from the CAG promoter group. **(D)** Idem as **(C)**, but for the hSyn promoter group. For **(C,D)**, the upper panels represent the 3-week-old neural culture, and the lower panel shows the 6-week-old neural culture. The images were acquired from an EVOS-FL microscope (scale bar = 200 μm).

To explore neuronal development and activity, neural cultures were effectively generated by following distinct stages, starting from iPSCs to neural cells (Figure 1A), using commercially defined protocols, culture media, and supplements (Figure 1B). Afterward, on DIV10 and DIV30, the neural cultures were virally infected with the AAV2/retro_hSyn_GCaMP6f and AAV2/retro_CAG_GCaMP6f constructs, and their fluorescence expression was regularly monitored. Figures 1C,D illustrates the results obtained from the two GECIs AAV2/retro_hSyn_GCaMP6f and AAV2/retro_CAG_GCaMP6f on weeks 3 and 6. Additionally, the CAG promoter groups showed both an earlier onset and a higher intensity of expression compared to the hSyn promoter groups, and all iPSC lines exhibited a similar pattern of fluorescence expression (Supplementary Figure S1). The targeted genes reached an optimum expression level around day 10 after the viral infection.

### Characterization of iPSC-derived neural culture and GECI expression level on week 3 and week 6

**Figure 2:**
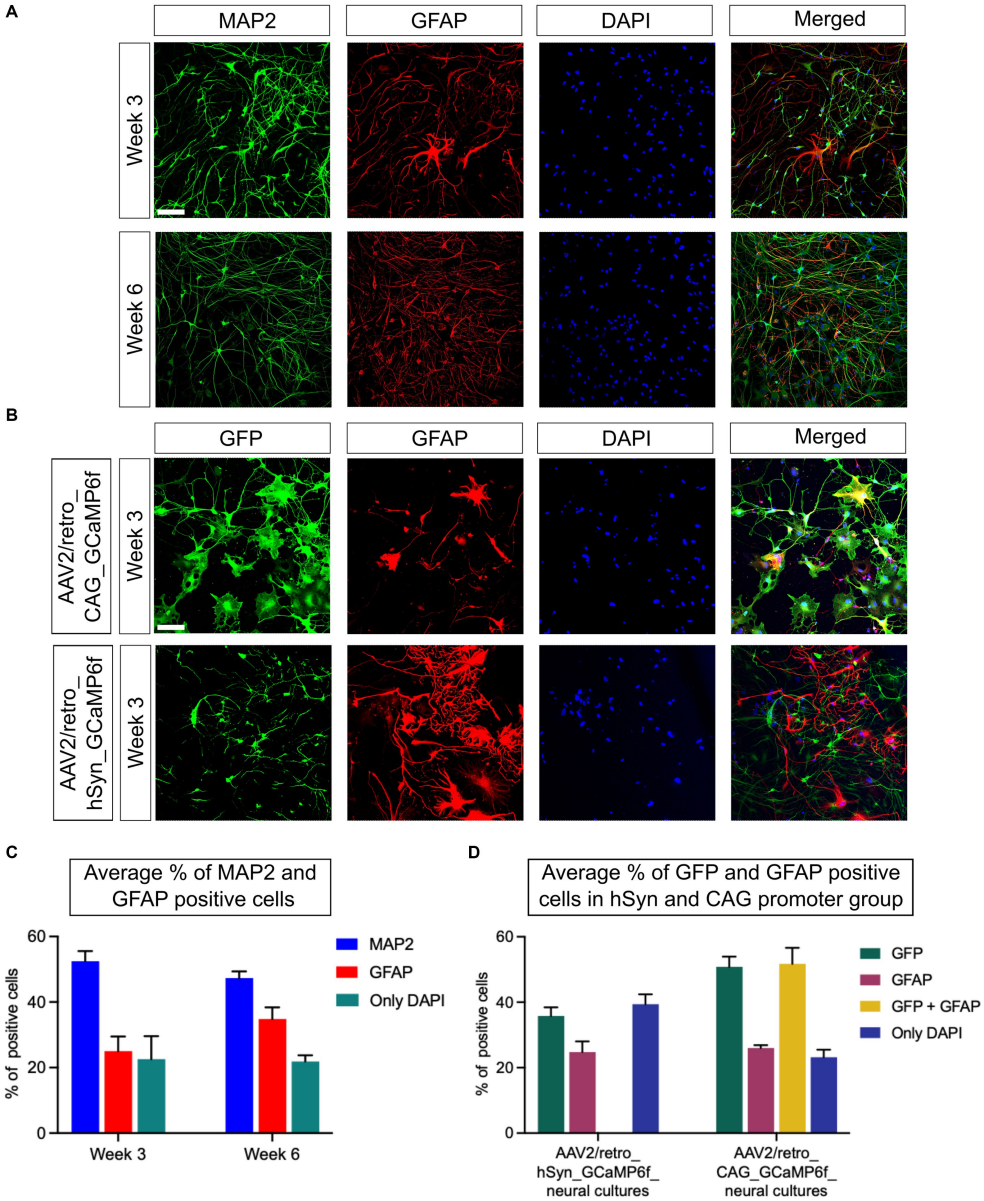
Optimization and characterization of neural culture from iPSCs. **(A)** The neural cells were differentiated from NPCs and immunostained with the neuronal marker MAP2 and the glial cell marker GFAP. The MAP2 (green) expression and GFAP (red) fluorescence levels were used to determine the ratio of targeted cells, while DAPI (blue) staining was used to determine the total number of cells in the field of view. The upper panel shows the expression of MAP2 and GFAP on week 3, and the lower panel on week 6. **(B)** Fluorescence images of neural cultures transfected with GCaMP6f are presented. A GFP tag marker was used to identify the cells transduced using either the hSyn or CAG promoters. The upper panel images illustrate the targeted protein expression of GFP (green), GFAP (red), and DAPI (blue) in the AAV2/retro_CAG_GCaMP6f promoter group on week 3. Similarly, the lower panel images represent the AAV2/retro_hSyn_GCaMP6f promoter group neural culture on week 3 (scale bar = 100 μm). **(C)** The graph shows the average neuronal and glial cell percentages from all three iPSC lines on week 3 and week 6 (*n* = 6 CSs/3 iPSC lines). No significant difference between the two time points was observed (*p* = 0.22). **(D)** The representative graph shows the percentage of GFP- and GFAP-positive cells in AAV2/retro_hSyn_GCaMP6f and AAV2/retro_CAG_GCaMP6f groups of neural culture relative to DAPI-positive cells on week 3. Moreover, the CAG promoter group showed colocalization between GFP and GFAP, whereas there was virtually no colocalization between GFP and GFAP markers in the hSyn promoter group for 3-week-old neural cultures (*n* = 6 CSs/3 iPSC lines). All data are presented as mean ± SEM.

As presented in the “Materials and methods” section, neural cultures were fixed on week 3 and week 6, on the same day of live neural cell Ca^2+^ imaging, to assess the neuron and glial cell ratio by immunostaining assay. The neurons were identified using the MAP2 protein marker, while the glial cells used the GFAP indicator. In addition, all nuclei were counterstained with DAPI (Figure 2A). The results show that the average percentage of the neuronal population was 52.5% on week 3, decreasing to 47.3% on week 6 [*p* = 0.24; difference between means (week 6 – week 3) ± SEM = −5.2 ± 3.7]. In contrast, the expression of GFAP was 24.9% on week 3, increasing to 34.7% on week 6 [*p* = 0.19; difference between means (week 6 – week 3) ± SEM = 9.8 ± 5.7]. Furthermore, the amount of only DAPI-positive cells was 22.6% on week 3 and 21.9% on week 6 [*p* = 0.9; difference between means (week 6 – week 3) ± SEM = −0.7 ± 7.2; n = 6 CSs/3 iPSC lines] (Figure 2C). The immunostaining with a GFP tag and GFAP marker has been performed on AAV2/retro_hSyn_GCaMP6f and AAV2/retro_CAG_GCaMP6f neural cultures to evaluate the neuronal cells expression efficiency and specificity of the hSyn promoter as well as the CAG promoter on week 3 (Figure 2B). The GFP- and GFAP-positive cells were assessed relative to DAPI staining (Figure 2D). In the AAV2/retro_hSyn_GCaMP6f group, the percentage of total positive cells was as follows: GFP 35.8% (SEM ± 2.6), GFAP 24.8% (SEM ± 2.2), and only DAPI 39.4% (SEM ± 3.0). In the hSyn promoter group, neural cultures did not show GFP and GFAP colocalization, stressing neuronal specificity, whereas the AAV2/retro_CAG_GCaMP6f group showed the following percentage of total positive cells: GFP 50.8% (SEM ± 3.1), GFAP 26.0% (SEM ± 0.9), and only DAPI 23.2% (SEM ± 2.3). The CAG promoter group demonstrated 51.7% (SEM ± 4.9) colocalization of GFP- and GFAP-positive neural cells (Figure 2D). The analysis indicated that the glial cell populations in both groups were quite similar, suggesting that the promoters had no significant effect on neural cell composition. Moreover, the CAG promoter was expressed in both neurons and glial cells, while the hSyn promoter showed high specificity for neurons.

### Characterization of hSyn and CAG promoter GECI expression in neurons

**Figure 3:**
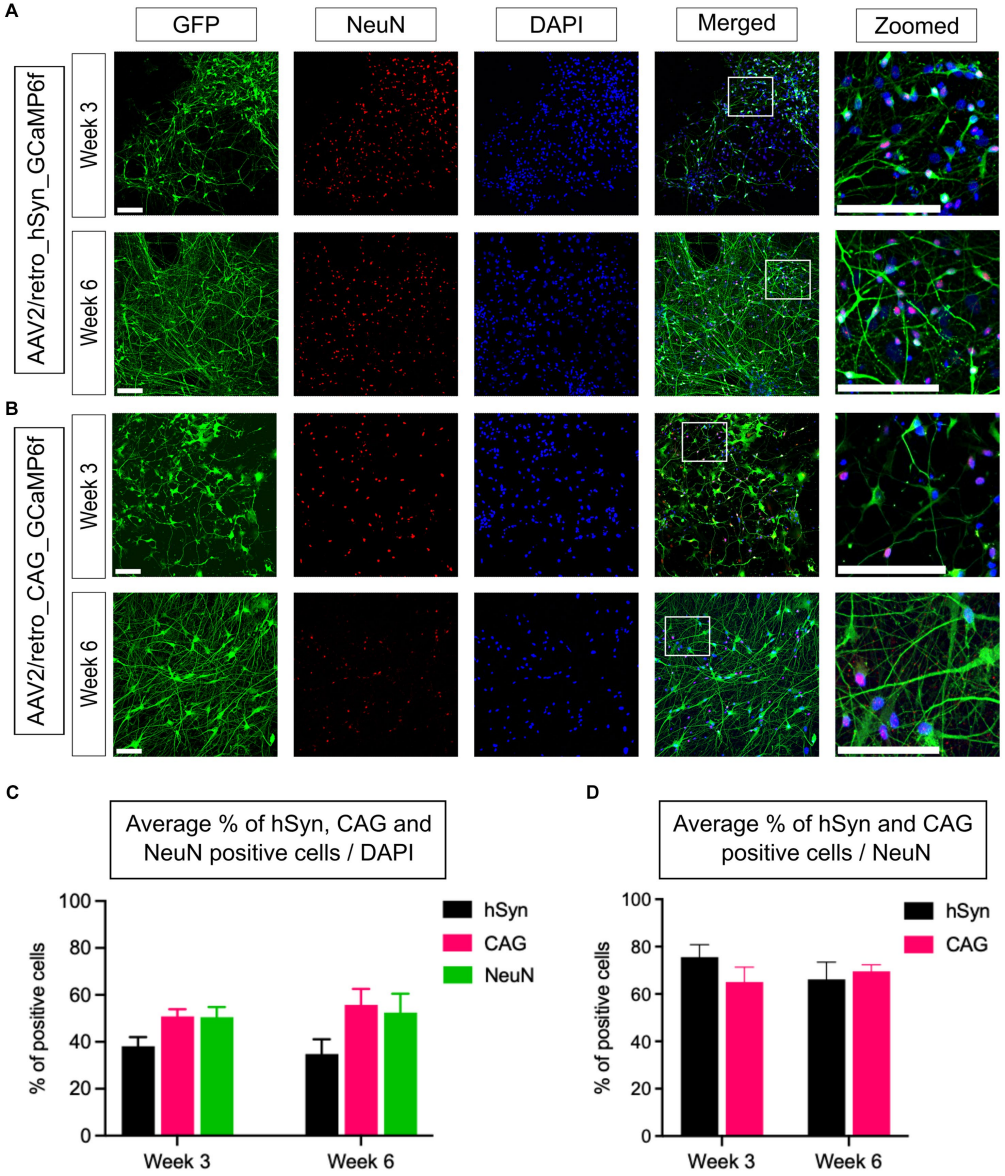
Quantification of hSyn and CAG promoter-based GCaMP6f expression for 3- and 6-week-old neurons *in vitro*. **(A)** Neural cultures were fixed and labeled to identify GFP-, NeuN-, and DAPI-positive cells. The upper panel illustrates the expression of GFP-positive cells in the 3-week-old, and the lower panel represents a 6-week-old neural culture transfected with AAV2/retro_CAG_GCaMP6f (scale bar = 200 μm) 10 days prior to imaging. **(B)** Neural cultures were transfected with AAV2/retro_hSyn_GCaMP6f, and immunoassays were performed using a GFP marker to identify the hSyn expression, a NeuN marker to identify neurons, and counterstaining using a DAPI marker for all cells. The upper panel represents 3-week-old neurons, whereas the lower panel represents 6-week-old neurons (scale bar = 200 μm). **(C)** The bar graph illustrates the mean percentage of GFP-positive cells (from hSyn and CAG promoter groups) and NeuN-positive cells relative to the total number of DAPI-stained nuclei from 3- and 6-week-old neural cultures. The statistical analysis showed no significant differences within or between the promoter groups (hSyn *p* = 0.68, CAG *p* = 0.56, and NeuN *p* = 0.84; *n* = 6 CSs/3 iPSC lines from each group). **(D)** The bar graph shows the average percentage of hSyn and CAG promoter groups GFP-positive cells relative to the total number of NeuN-positive cells (hSyn vs. CAG; week 3, *p* = 0.27; week 6, *p* = 0.68; *n* = 6 CSs/3 iPSC lines from each group).

To assess the efficiency of GECI expression induced by hSyn and CAG promoters in neurons, an immunostaining assay was conducted using a GFP tag to identify cells expressing the hSyn or CAG promoter, a NeuN marker to identify neurons, and counterstaining with DAPI to stain all cell nuclei. Under similar conditions, immunostained neural cultures were imaged and analyzed, and representative images of the hSyn promoter (Figure 3A) and CAG promoter (Figure 3B) groups’ neural cultures expressing GFP- and NeuN-positive cells were obtained. The statistical analysis within and between groups showed no significant difference in the expression level of the hSyn promoter group on week 3 (38.1%) and week 6 (34.8%) [*p* = 0.68; difference between means (week 6 - week 3) ± SEM = −3.3 ± 7.4]. The CAG promoter group also did not show a difference between week 3 (50.8%) and week 6 (55.7%) [*p* = 0.54; difference between means (week 6 – week 3) ± SEM = 4.9 ± 7.5]. Furthermore, the percentage of NeuN-positive cells on week 3 (50.5%) did not show a difference from week 6 (52.5%) [*p* = 0.84; difference between means (week 6 - week 3) ± SEM = 1.9 ± 9.2] (Figure 3C). In the hSyn and CAG promoter groups, GFP-positive cells were compared with NeuN-positive cells. The hSyn promoter group showed 75.6% of neurons were positive with GFP on week 3, whereas 66.2% of neurons were positive on week 6 [*p* = 0.35; difference between means (week 6 – week 3) ± SEM = −9.4 ± 9.0] (Figure 3D). Furthermore, the CAG promoter group analysis showed 65.1% of neurons were positive on week 3 and 69.6% of neurons were positive with GFP on week 6 [*p* = 0.54; difference between means (week 6 – week 3) ± SEM = 4.5 ± 6.9] (Figure 3D). Notably, there were no significant differences between hSyn and CAG promoter GECI expression on neurons at both time points of analysis (Figure 3D). Therefore, this result suggests that hSyn- and CAG-promoters of GECI express similarly in iPSC-derived neurons.

### Neuronal Ca^2+^ imaging analysis workflow

**Figure 4:**
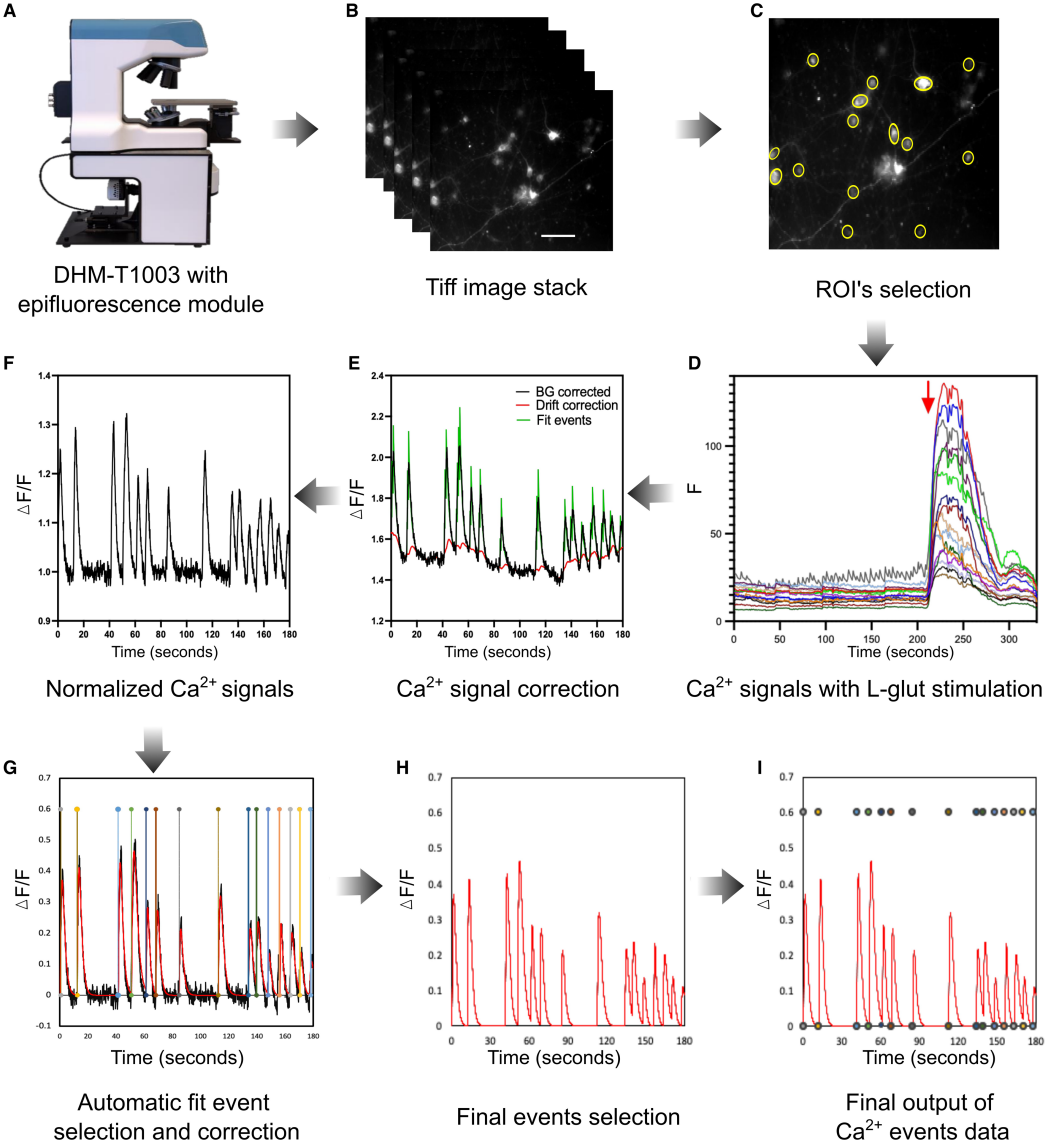
Live neuronal Ca^2+^ imaging setup and analysis workflow. **(A)** A customized multimodal epifluorescence microscope (DHM-T1003, Lyncée Tec) was used to monitor live neuronal Ca^2+^ spontaneous events. **(B)** A representative TIFF image stack was acquired with a magnification of 20× (0.70 NA) and 1,329 × 1,080 pixels resolution. **(C)** MATLAB image processing pipeline was used to identify neuronal soma as ROIs and imported them into the ImageJ software to obtain Ca^2+^ intensity data. **(D)** Example of background-subtracted spontaneous neuronal cell Ca^2+^ activity with a short application of L-glut (30 μM, 30 s) to identify neurons. **(E,F)** The normalization of neuronal spontaneous Ca^2+^ events was carried out with a MATLAB algorithm, which is utilized to correct background and signal drift and identify fit events. **(G,H)** In the next step, the pipeline automatically detects events to evaluate the signal parameters as mentioned in the “Results” section (red line). In this step, all Ca^2+^ signals were also manually reviewed and corrected if necessary to avoid any false Ca^2+^ detection. **(I)** The illustration depicts the final corrected neuronal spontaneous Ca^2+^ events for individual cells. The Ca^2+^ events were included in the analysis if their amplitude was two times higher than the standard deviation of the baseline level.

The neuronal Ca^2+^ activity assessment was divided into five main steps: acquisition and saving (Figures 4A,B), selection of the regions of interest (ROIs), data processing, Ca^2+^ spontaneous event peak identification, and estimation of peak parameters. Specifically, a custom-made algorithm written in MATLAB (version 9.1, MathWorks) was designed to detect the cell soma based on maximum intensity fluorescence, threshold the image, and generate an image delineating the ROIs that can be loaded in ImageJ using the ROI manager. Further automated ROI selections were manually verified and validated to prevent incorrect selections or to add any missing cells if needed (Figure 4C). To adjust the baseline signal of cellular data, multiple background ROIs (areas without cells) were also manually defined within the field of view. The raw ROI intensity traces were then exported back into MATLAB. The intensity time traces for each of the ROIs selected were then stored in comma-separated values (CVS) files. The time traces were analyzed using another MATLAB script adapted from Deneux et al. (2016) that applied a background correction, normalized and smoothed the data, and detected Ca^2+^ events. Concretely, this data processing was used to subtract the background, correct the signal for potential drift or photobleaching, and detect spontaneous Ca^2+^ events.

First, the background correction was performed, and each trace baseline fluorescence level was calculated as the average basal fluorescence intensity. The Ca^2+^ signal intensity of each raw trace was then normalized by calculating Δ*F/F* as the ratio of the increase in fluorescence (Δ*F*) to the baseline (*F*). Next, the signal drift, background, and baseline corrected data were saved for further analysis (Figures 4E,F). In the final step, an automatic fit event correction was performed to identify the Ca^2+^ spontaneous events. Additionally, all the automatic Ca^2+^ signal fit events were manually reviewed and corrected if necessary. The neuronal Ca^2+^ spontaneous activity was considered a signal if it was ≥2× the baseline mean standard deviation values (Figures 4G–I). Each data set was evaluated under identical experimental conditions as described earlier during the analysis process. The last step consists of estimating single-event parameters. To achieve this, each detected Ca^2+^ event was fitted using the following 4-parameter equation:


It=Ampτdecay−τrisee−t−t0τdecay−e−t−t0τrise,

where Amp , $\tau_{\mathrm{decay}}$ , $\tau_{\mathrm{rise}}$ , and $t_{0}$ represent, respectively, the amplitude, the decay time, the rise time, and the time of each event. The parameters were exported in xlsx files for comparison.

Practically, 180-s Ca^2+^ trace data were considered to evaluate the neuronal network activity. After 180 s of live Ca^2+^ imaging, L-glut was applied to the neural culture to identify the neurons (Figure 4D). Only cells that had a clear response to L-glut were kept in the analysis (Figure 4D) and were assumed to be neurons (Supplementary Figure S2). Indeed, L-glut has been frequently used by researchers in live Ca^2+^ imaging experiments to recognize the neuronal cells in mixed cortical cultures *in vitro*. Specifically, L-glut application induces substantial Ca^2+^ influx in the neurons due to the vast number of L-glut-responsive receptors, mainly NMDA receptors, in comparison to non-neuronal cells, including glial cells such as astrocytes which may show only a transient Ca^2+^ influx (Pickering et al., 2008).

### Evaluation of neuronal spontaneous Ca^2+^ events

**Figure 5:**
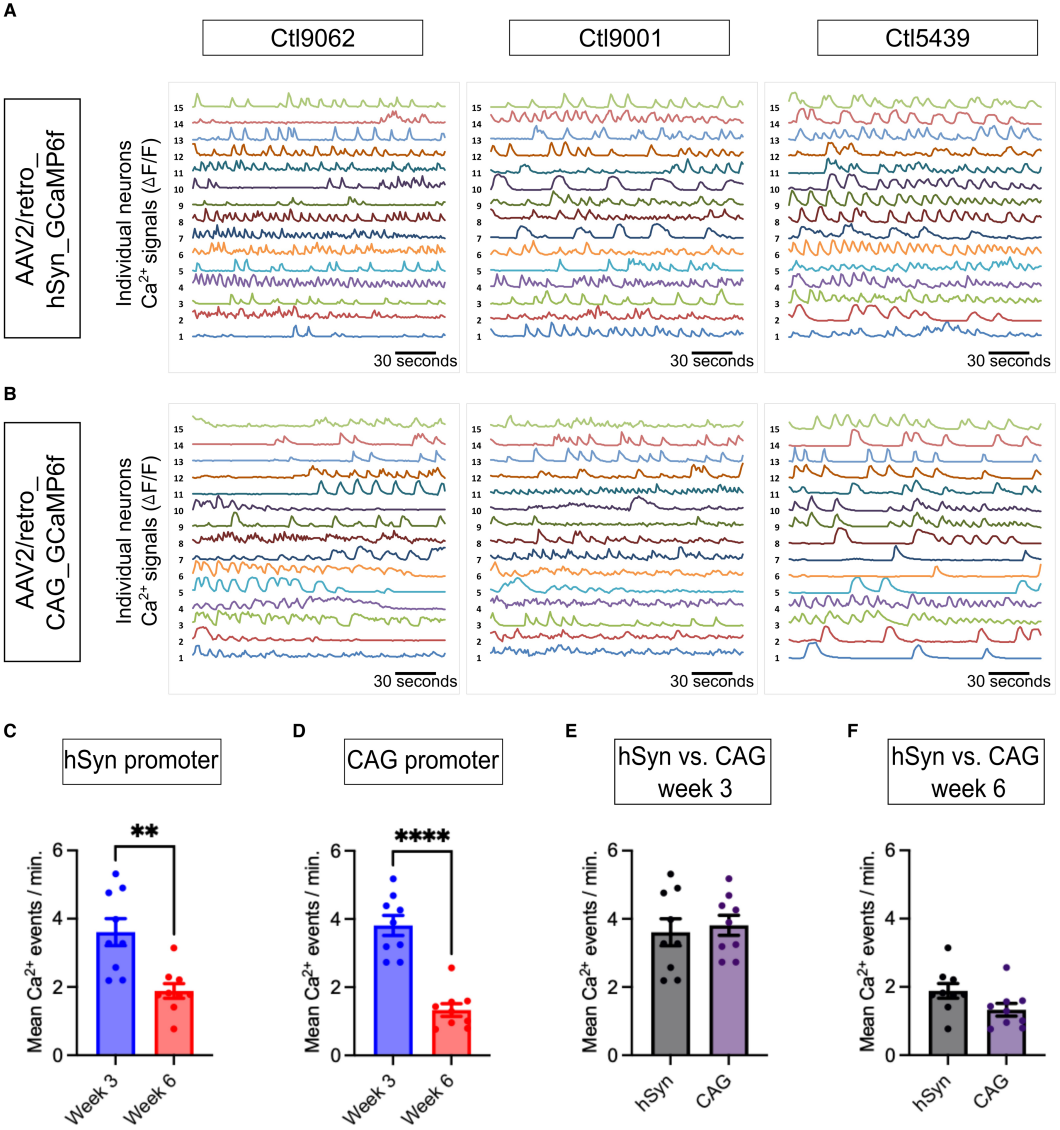
Neuronal network activity and analysis of neuronal Ca^2+^ dynamics. **(A,B)** Normalized spontaneous Ca^2+^ events plotting fluorescent intensity for 180 s at a 10 Hz frame rate are presented. The representative spontaneous Ca^2+^ events are presented from three iPSC lines (Ctl9062, Ctl9001, and Ctl5439; Y-axis, with each number representing the Ca^2+^ events of single neurons in ∆*F/F* form a single field of view on week 3). **(C)** The bar graph presents the mean number of Ca^2+^ events per minute from the hSyn promoter group, which shows a significant increase on week 3 compared to week 6 (*p* = 0.002). **(D)** The bar graph illustrates the average number of Ca^2+^ events per minute in the CAG promoter group. The total Ca^2+^ events show a significant increase on week 3 compared to week 6 (*p* = 0.0001). **(E,F)** The bar graph represents the mean number of Ca^2+^ events between the hSyn and CAG promoter groups (on week 3, *p* = 0.685 and on week 6, *p* = 0.071). The black dots on the bar graph from **(C)** to **(F)** represent the average number of Ca^2+^ events obtained from individual CS across all three iPSC lines. All Ca^2+^ data are represented as mean ± SEM (*p < 0.05, **p < 0.01, and ***p < 0.001).

The spontaneous Ca^2+^ events in neurons were assessed and compared between the hSyn and CAG promoter groups. The representative Ca^2+^ traces from all three iPSC lines (Ctl9062, Ctl9001, and Ctl5439) show neuronal spontaneous activity in 3-week-old neurons (Figures 5A,B). The neuronal Ca^2+^ intensity signals, represented as Δ*F*/*F* values, for the hSyn promoter group (Figure 5A) and the CAG promoter group (Figure 5B), illustrate the neuronal Ca^2+^ events of individual neurons within a single field of view. Notably, our findings depict that neuronal Ca^2+^ spontaneous events significantly decline over time. In the hSyn promoter group, neurons showed higher spontaneous activity at 3.61 events per minute on week 3, which declined to 1.89 events per minute on week 6 [*p* = 0.002; difference between means (week 6 – week 3) ± SEM = −1.72 ± 0.45] (Figure 5C). The CAG promoter group also showed a similar trend. On week 3, spontaneous neuronal Ca^2+^ activity was 3.81 events per minute, whereas on week 6, it was 1.33 events per minute [*p* = 0.0001; difference between means (week 6 – week 3) ± SEM = −2.48 ± 0.35] (Figure 5D). However, no significant differences were found within the three iPSC lines at both time points in both virally infected groups (data not shown). Conversely, when hSyn and CAG promoter groups were compared on week 3 [*p* = 0.69; difference between means (hSyn week 3 – CAG week 3) ± SEM = 0.20 ± 0.49] and on week 6 [*p* = 0.07; difference between means (hSyn week 6 – CAG week 6) ± SEM = −0.55 ± 0.29], no significant differences can be observed (Figures 5E,F). Hence, our data confirmed that spontaneous neuronal network activity is higher in the early phase (week 3) of neuronal development compared to the late phase (week 6). Furthermore, both hSyn and CAG promoter-based GCaMP6f enabled accurate assessment of the spontaneous frequency of Ca^2+^ in neuronal cells. We also tested TTX, a blocker of Na^+^ channels, in some experiments to validate that spontaneous Ca^2+^ events are mainly associated with Na^+^ channels (Supplementary Figure S2). We observed that in the presence of TTX, spontaneous Ca^2+^ events were significantly reduced or abolished, and they reappeared after washing it out (Supplementary Figure S2), which suggests that Ca^2+^ events presumably result from voltage changes through the depolarization of neurons (Chong and Ruben, 2008).

### Assessment of neuronal spontaneous Ca^2+^ event kinetics

**Figure 6:**
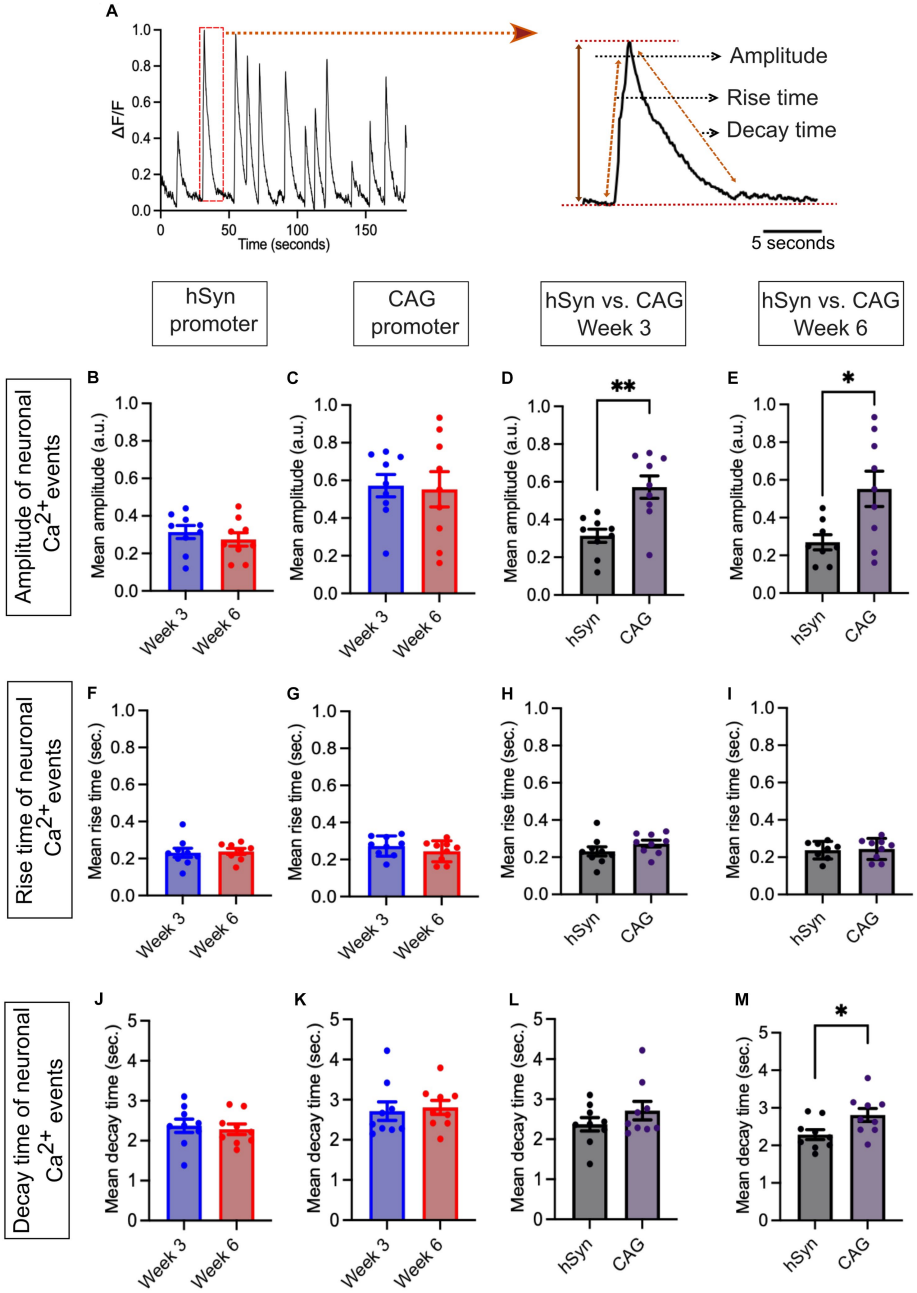
Ca^2+^ spontaneous activity for hSyn and CAG promoter groups. **(A)** Representative normalized Ca^2+^ traces (left) and estimated parameters were obtained using a laboratory-designed MATLAB algorithm (right). The parameters for each event were obtained using the equation: It=Ampτdecay−τrisee−t−t0τdecay−e−t−t0τrise , where Amp, $\tau_{\mathrm{decay}}$ , $\tau_{\mathrm{rise}}$ , and $t_{0}$ represent the amplitude, the decay time, the rise time, and the time of the event, respectively. **(B)** The graph shows the mean amplitude of neuronal Ca^2+^ events obtained from the hSyn promoter group neural culture on FIGURE 6 (Continued)week 3 and week 6 (*p* = 0.439). **(C)** The graph represents the mean amplitude of neuronal Ca^2+^ events from the CAG promoter group neural culture on week 3 and week 6 (*p* = 0.864). **(D,E)** The mean amplitude bar graph of neuronal Ca^2+^ events showed significantly lower values in the hSyn promoter group in contrast to the CAG promoter group on week 3 (*p* = 0.002) as well as on week 6 (*p* = 0.02). **(F)** The neuronal Ca^2+^ event mean rise time of hSyn promoter GECI did not differ much at both time points (week 3 and week 6; *p* = 0.82). **(G)** The neuronal Ca^2+^ events mean rise time of CAG promoter GECI was not different at both time points (week 3 and week 6; *p* = 0.29). **(H,I)** The bar graph illustrating the mean rise time of Ca^2+^ events indicated no significant difference between the hSyn and CAG promoter groups at both time points, week 3 (*p* = 0.191) and week 6 (*p* = 0.815). **(J)** The mean decay time bar graph of neuronal Ca^2+^ events in the hSyn promoter group did not differ between week 3 and week 6 (*p* = 0.69). **(K)** The bar graph of the mean decay time of the CAG promoter group. No differences were found within the group on week 3 and week 6 (*p* = 0.75). **(L,M)** On week 3, the mean decay time (*p* = 0.249) did not exhibit significant differences. However, on week 6, significantly higher decay times in the CAG promoter group were observed (*p* = 0.03). The black dots on the bar graph from **(C)** to **(F)** represent the average Ca^2+^ events obtained from individual CS across all three iPSC lines. All Ca^2+^ data are represented as mean ± SEM (*p < 0.05, **p < 0.01, and ***p < 0.001).

The neuronal spontaneous Ca^2+^ events were further analyzed and compared in terms of amplitude, rise time, and decay time of signals (Figure 6A) obtained from the hSyn and CAG promoter groups. The amplitudes of spontaneous Ca^2+^ events were compared within and between both groups on week 3 and week 6. Within the hSyn promoter group, the average amplitude of spontaneous Ca^2+^ events was 0.31 a.u. on week 3 and 0.28 a.u. on week 6 [*p* = 0.439; difference between means (week 6 – week 3) ± SEM = −0.4 ± 0.5] (Figure 6B), while in the CAG promoter group, the average amplitude was 0.57 a.u. at week 3 and 0.55 a.u. at week 6 [*p* = 0.864; difference between means (week 6 – week 3) ± SEM = −0.02 ± 0.11] (Figure 6C). Although there were no significant differences in Ca^2+^ event amplitude within each group between week 3 and week 6, the hSyn promoter group displayed a significantly lower average amplitude at both time points. On week 3, the hSyn promoter group amplitude was 0.31 a.u., whereas the CAG promoter group amplitude was 0.57 a.u. [*p* = 0.002; difference between means (hSyn week 3 – CAG week 3) ± SEM = 0.26 ± 0.07] (Figure 6D). At week 6, the hSyn promoter group amplitude was 0.28 a.u. and the CAG promoter group amplitude was 0.55 a.u. [*p* = 0.02; difference between means (hSyn week 6 – CAG week 6) ± SEM = 0.28 ± 0.11] (Figure 6E). In contrast, the rise time of spontaneous Ca^2+^ events was similar in both groups and showed no significant change between the two time points. The hSyn promoter group rise time was 0.23 s on week 3 and 0.24 s on week 6 [*p* = 0.82; difference between means (week 6 – week 3) ± SEM = 0.01 ± 0.03] (Figure 6F). Similarly, the CAG promoter group rise time was 0.27 s on week 3 and 0.24 s on week 6 [*p* = 0.29; difference between means (week 6 – week 3) ± SEM = −0.03 ± 0.03] (Figure 6G). The spontaneous Ca^2+^ event rise time between hSyn and CAG promoter groups did not differ from each other on week 3 [*p* = 0.19; difference between means (hSyn week 3 – CAG week 3) ± SEM = 0.04 ± 0.03] (Figure 6H) and on week 6 [*p* = 0.82; difference between means (hSyn week 6 – CAG week 6) ± SEM = 0.01 ± 0.03] (Figure 6I). The decay time of spontaneous Ca^2+^ events within groups did not show differences between the two time points in either group. In the hSyn promoter group, the decay time was 2.37 s on week 3 and 2.29 s on week 6 [*p* = 0.69; difference between means (week 6 – week 3) ± SEM = −0.09 ± 0.21] (Figure 6J). Meanwhile, in the CAG promoter group, the decay time was 2.71 s on week 3 and 2.81 s on week 6 [*p* = 0.75; difference between means (week 6 – week 3) ± SEM = 0.09 ± 0.29] (Figure 6K). Moreover, there were no differences in the decay time between the hSyn and CAG promoter groups on week 3 [*p* = 0.25; difference between means (hSyn week 3 – CAG week 3) ± SEM = 0.34 ± 0.28] (Figure 6L). However, on week 6, the hSyn and CAG promoter groups showed a significant difference in decay time [*p* = 0.03; difference between means (hSyn week 6 – CAG week 6) ± SEM = 0.52 ± 0.22] (Figure 6M). The neuronal spontaneous Ca^2+^ event kinetics data suggest that the properties of the Ca^2+^ events remained relatively stable within the hSyn and CAG promoter groups throughout the studied neurodevelopmental time period, despite the overall changes in Ca^2+^ event frequency. Furthermore, these findings indicate that the CAG promoter may lead to more robust Ca^2+^ signaling compared to the hSyn promoter in the studied neuronal population.

## Discussion

Our mixed cultures of iPSC-derived cortical neurons and astrocytes, obtained by reprogramming urothelial cells from healthy subjects, showed good reproducibility between the three differentiated lines, all resulting in the generation of highly homogeneous NPCs prior to differentiation (Figures 1A,B). Immunostaining results showed that, relative to the total cell population, 50%–60% are neurons and 20%–30% are glial cells (Figures 2A,C). These results are consistent with previously published results using the same differentiation protocol (Bardy et al., 2015). The remaining 20% of DAPI-only labeled cells may represent other cell types, including fibroblasts, endothelial cells, or cells of the astrocytic and/or neuronal lineages that have not reached a sufficient degree of maturation. Assuming that these 20% of other or insufficiently mature cells were not transfected and given that no GFP-positive cells colocalizing with the GFAP marker could be detected in the hSyn promoter group, this means that the hSyn promoter would have labeled more than 70% of the neuronal population. Under the same assumption, the CAG promoter would have labeled more than 60% of the astrocytes and neurons. This assumption is supported by the results in Figure 3D, which confirm that the hSyn promoter labels more than 70% of the neuronal population. Furthermore, Figure 3D shows that the CAG promoter targeted about 65% of the neuronal population, and considering that it targeted approximately 60% of the astrocytic and neuronal populations as assumed above, we should obtain a labeling of more than 50% of the astrocytic population, which is confirmed by Figure 2D. Furthermore, our immunostaining results were consistent with the previous study of hSyn in mouse models (Nathanson et al., 2009), confirming that hSyn was not only neuron-specific but also highly expressed in neurons.

After the validation of the molecular expression level, we further evaluated the neural network activity by analyzing the Ca^2+^ signals obtained from the hSyn and CAG promoter GECI using the analysis workflow described earlier (Figures 4C–I). This Ca^2+^ signal processing resulted in stable traces, making it easy to identify and quantitatively analyze spontaneous Ca^2+^ events. Overall, the neuronal Ca^2+^ traces of the three iPSC lines, although there were some differences, were quite similar. Furthermore, there was no significant difference between the hSyn and CAG groups; both showed similar patterns of spontaneous Ca^2+^ activity.

Our culture conditions and methodological approach showed similar Ca^2+^ event frequency levels in the early stages of the neurodifferentiation process at week 3, as previously reported with other chemical dye Ca^2+^ indicator protocols (Kuijlaars et al., 2016). However, our results at week 6 do not show more sustained activity in terms of Ca^2+^ events compared to those at week 3. On the contrary, a decrease in Ca^2+^ event activity was measured in week 6. Notably, although the neuronal Ca^2+^ events changed over time, the properties of these events, including rise time, decay time, and amplitude, exhibited a relatively consistent pattern between weeks 3 and 6. Since the firing rate is a proxy for the proper maturation of a functional network, these results may seem surprising. Although our immunostaining showed no significant loss of neurons after 6 weeks of culture, we cannot completely rule out the possibility that the good health of the neurons was beginning to be compromised by these prolonged culture conditions to explain these results. However, another possible reason for the reduced firing rate after 6 weeks of culture is the maturation of inhibitory systems within the network. Indeed, it has been shown in neuronal cultures that inhibition of GABAergic transmission increases neuronal activity, and consequently, GABA-releasing interneurons effectively inhibit neuronal firing (Eisenman et al., 2015). This argument is also supported by two studies showing that the addition of interneurons to pure glutamatergic neuron cultures derived from iPSCs reduced firing rate (Mossink et al., 2022; Parnell et al., 2023).

On the other hand, it should also be noted that even though GECI expression based on the hSyn promoter is weaker than that obtained with the CAG promoter, it does not prevent reliable Ca^2+^ imaging, enabling spontaneous Ca^2+^ events to be identified and analyzed. Furthermore, although the use of the non-neuron-specific CAG promoter enables neuronal Ca^2+^ activity to be properly monitored, these results are highly dependent on the use of L-glut perfusion to ensure that responses from neuronal cells are selected.

## Conclusion

This study presented an efficient and reliable methodology using GECI for multiple time point Ca^2+^ imaging during the neurodifferentiation of human iPSCs into different types of cortical neurons and astrocytes. This required the testing of multiple serotype variants to obtain viral constructs that produced appropriate levels of GECI expression. For validation purposes, the AAV2/retro_GCaMP6f variant was used in combination with the hSyn promoter, known for its neuron-specific expression, and compared with the ubiquitously expressed CAG promoter. Specifically, neuronal Ca^2+^ activity was monitored under similar conditions with AAV2/retro_hSyn_GCaMP6f and AAV2/retro_CAG_GCaMP6f.

To achieve this monitoring, an efficient procedure for the analysis of Ca^2+^ signals was made possible by the development of a multi-step algorithm that allows the discrimination and quantitative analysis of spontaneous events in a simple, semi-automatic way. According to this methodology, the hSyn promoter, despite having lower expression levels than the CAG promoter, proved to be very suitable to specifically measure neuronal Ca^2+^ activities at different time points, which were compatible with the neurodifferentiation process. In our opinion, this methodology is therefore robust and reliable for monitoring neuronal Ca^2+^ activity, especially spontaneous events, throughout the neurodifferentiation process in mixed cultures containing astrocytes and cortical neurons, a highly relevant *in vitro* cell model for studying the pathogenesis and pathophysiology of neuropsychiatric diseases. This methodology may also be relevant for compound screening in the context of drug discovery as these iPSC-derived neuronal cultures can be generated not only from control subjects but also from patients with neuropsychiatric disorders.

## Data availability statement

The original contributions presented in the study are included in the article/Supplementary material, further inquiries can be directed to the corresponding author.

## Ethics statement

The studies involving humans were approved by CIUSSS-CN 555, boul. Wilfrid-Hamel Québec (Québec) G1M 3X7, Canada. The studies were conducted in accordance with the local legislation and institutional requirements. The participants provided their written informed consent to participate in this study.

## Author contributions

NP designed the study plan, performed all experiments, analyzed the data, drafted the manuscript, and designed the figures. VO performed the computational framework in MATLAB to analyze the Ca^2+^ data. FP-M developed the operating software, built the acquisition electronics of the microscope, and automated the external devices involved in the experimental protocols. NC and M-EP designed, manufactured, and delivered all GECI viral vector samples. EB adapted the optics of the epifluorescence microscope and designed the perfusion chambers, advised on the microscopic study, and assisted in the correction of the manuscript. AGG implemented the computational framework in MATLAB to analyze the Ca^2+^ data, verified the numerical results, assisted in the interpretation of the results, and assisted in manuscript correction. PM developed the main conceptual ideas and supervised the entire study. All authors contributed to the article and approved the submitted version.

## Funding

This study was supported by the Canada Excellence Research Chairs Program, the Canada Foundation for Innovation (34265, 36689), the Natural Sciences and Engineering Research Council of Canada (RGPIN-2018-06198), the Neuro-CERVO Alliance for Drug Discovery in Brain Diseases and the International Joint Unit on Neurodevelopment and Child Psychiatry between *Université Laval*, Canada, and *Université de Lausanne*, Switzerland (PM). AGG is a Scholar of the *Fonds de recherche du Québec—Santé* and was supported by a Sentinel North Partnership Research Chair and grant #06507 from the Natural Sciences and Engineering Research Council of Canada. The viral work (M-EP) is supported by the Brain Canada Foundation (Platform Support Grant).

## Conflict of interest

PM declares a potential conflict as co-founder of Lyncée Tec. However, this independent study was performed in academic laboratories.

The remaining authors declare that the research was conducted in the absence of any commercial or financial relationships that could be construed as a potential conflict of interest.

## Publisher’s note

All claims expressed in this article are solely those of the authors and do not necessarily represent those of their affiliated organizations, or those of the publisher, the editors and the reviewers. Any product that may be evaluated in this article, or claim that may be made by its manufacturer, is not guaranteed or endorsed by the publisher.
