# Supplementary material for: A robust and reliable methodology to perform GECI-based multi-time point neuronal calcium imaging within mixed cultures of human iPSC-derived cortical neurons
Source: Front Neurosci. 2023 Sep 25;17:1247397. doi: 10.3389/fnins.2023.1247397 (PMC10560759; doi:10.3389/fnins.2023.1247397)
Supplement: Supplementary file 1 [file Data_Sheet_1.docx]

Supplementary Material

# A robust and reliable methodology to perform GECI-based multi-time point neuronal calcium imaging within mixed cultures of human iPSC-derived cortical neurons

Niraj Patel, Vincent Ouellet, François Paquet-Mercier, Nizar Chetoui, Erik Bélanger, Marie-Eve Paquet, Antoine G. Godin, and Pierre Marquet^*^

***Correspondence:**

Pierre Marquet

[pierre.marquet@neuro.ulaval.ca](mailto:pierre.marquet@neuro.ulaval.ca)

**Supplementary Table S1.** List of serotypes tested on iPSC-derived neural cultures.

| **No.** | **Name of serotypes constructs** | **Concentration** |
| --- | --- | --- |
| 1 | AAV2/retro-hSyn-GCaMP6f | 1x10^10^ GC/mL |
| 2 | AAV2/retro-hSyn-GCaMP6f | 1x10^10^ GC/mL |
| 3 | AAV2/retro-hSyn-GFP | 1x10^10^ GC/mL |
| 4 | AAV2/9-hSyn-GCaMP6f | 1x10^10^ GC/mL |
| 5 | AAV2/retro-CAG-eGFP | 1x10^10^ GC/mL |
| 6 | AAV2/retro-CAG-GCaMP6f | 1x10^10^ GC/mL |
| 7 | AAV2/php.S-CAG-eGFP | 1x10^10^ GC/mL |
| 8 | AAV2/php.eB-CAG-eGFP | 1x10^10^ GC/mL |
| 9 | AAV2/Po6-CAG-eGFP | 1x10^10^ GC/mL |
| 10 | AAV2/DJ8-CAG-eGFP | 1x10^10^ GC/mL |
| 11 | AAV2/DJ-CAG-eGFP | 1x10^10^ GC/mL |
| 12 | AAV2/RH10-CAG-eGFP | 1x10^10^ GC/mL |
| 13 | AAV2/9-CAG-eGFP | 1x10^10^ GC/mL |
| 14 | AAV2/8-CAG-eGFP | 1x10^10^ GC/mL |
| 15 | AAV2/6-CAG-eGFP | 1x10^10^ GC/mL |
| 16 | AAV2/5-CAG-eGFP | 1x10^10^ GC/mL |
| 17 | AAV2/2-CAG-eGFP | 1x10^10^ GC/mL |
| 18 | AAV2/1-CAG-eGFP | 1x10^10^ GC/mL |

Abbreviation: AAV, adeno-associated virus; eGFP, enhanced green fluorescent protein.

**Supplementary Figure S1.** The viral infection and expression of hSyn and CAG promoter groups in iPSC-derived neural cultures.

**
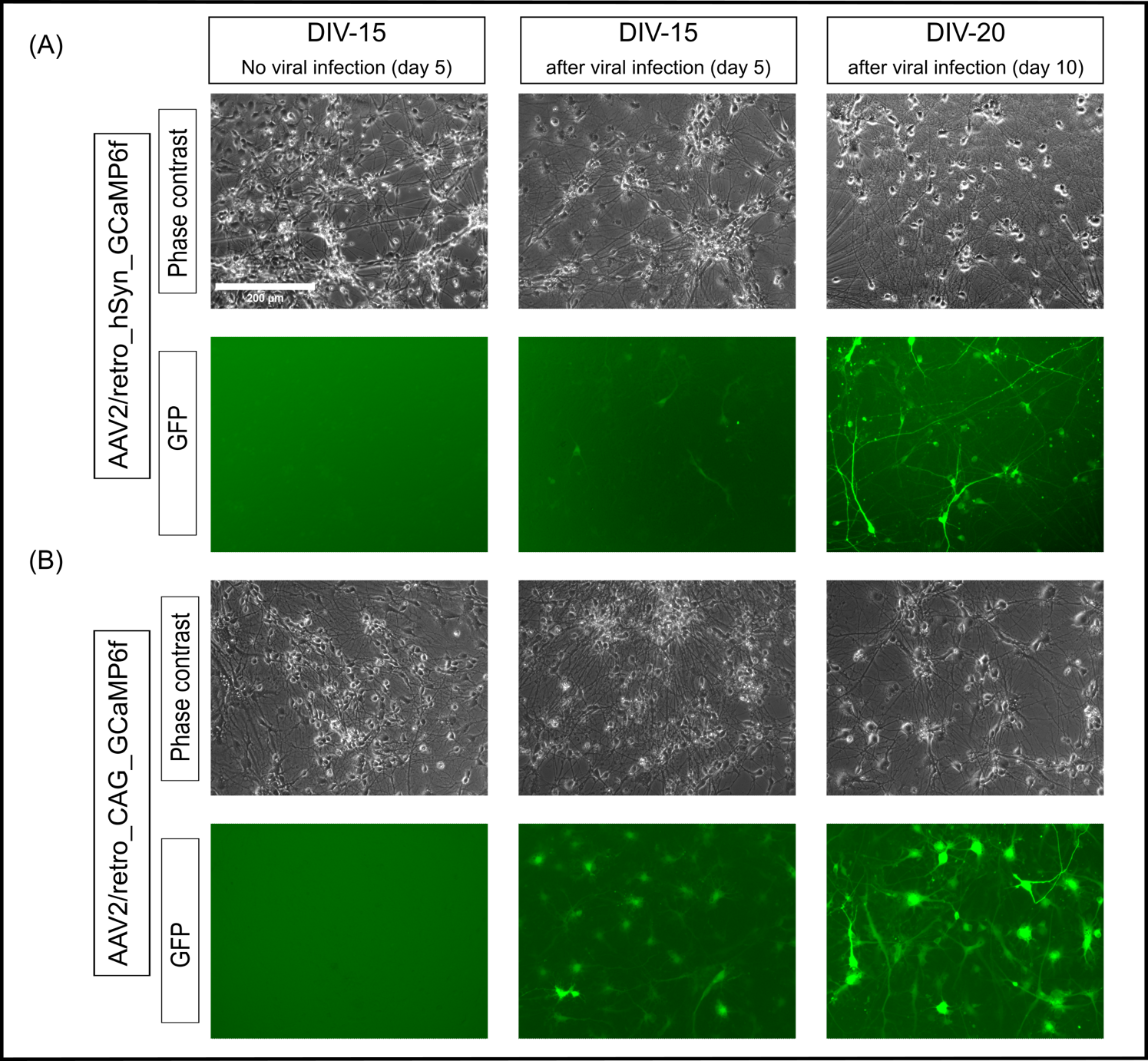
**

The neural cultures were infected with AAV2/retro_hSyn_GCaMP6f or AAV2/retro_CAG_GCaMP6f on DIV-10 and were visually monitored on alternate days. The images were captured using an EVOS-FL microscope at a 20X objective on DIV-15 and DIV-20. (A) Representative images display the phase contrast (upper panel) and fluorescence (lower panel). The left images represent the positive control with no viral infection. The middle images show the hSyn expression level on DIV-15 (5 days after viral infection), while the right images depict the hSyn expression on DIV-20 (10 days after viral infection). (B) Representative images illustrate the phase contrast (upper panel) and fluorescence (lower panel). The left images represent the positive control with no viral infection. The middle images exhibit the hSyn expression level on DIV-15 (5 days after viral infection), while the right images depict the hSyn expression on DIV-20 (10 days after viral infection) (scale bar = 200 μm).

**Supplementary Figure S2.** The neuronal Ca^2+^ activity arises from voltage-gated calcium channels, representing the overall functionality of the neuronal network.


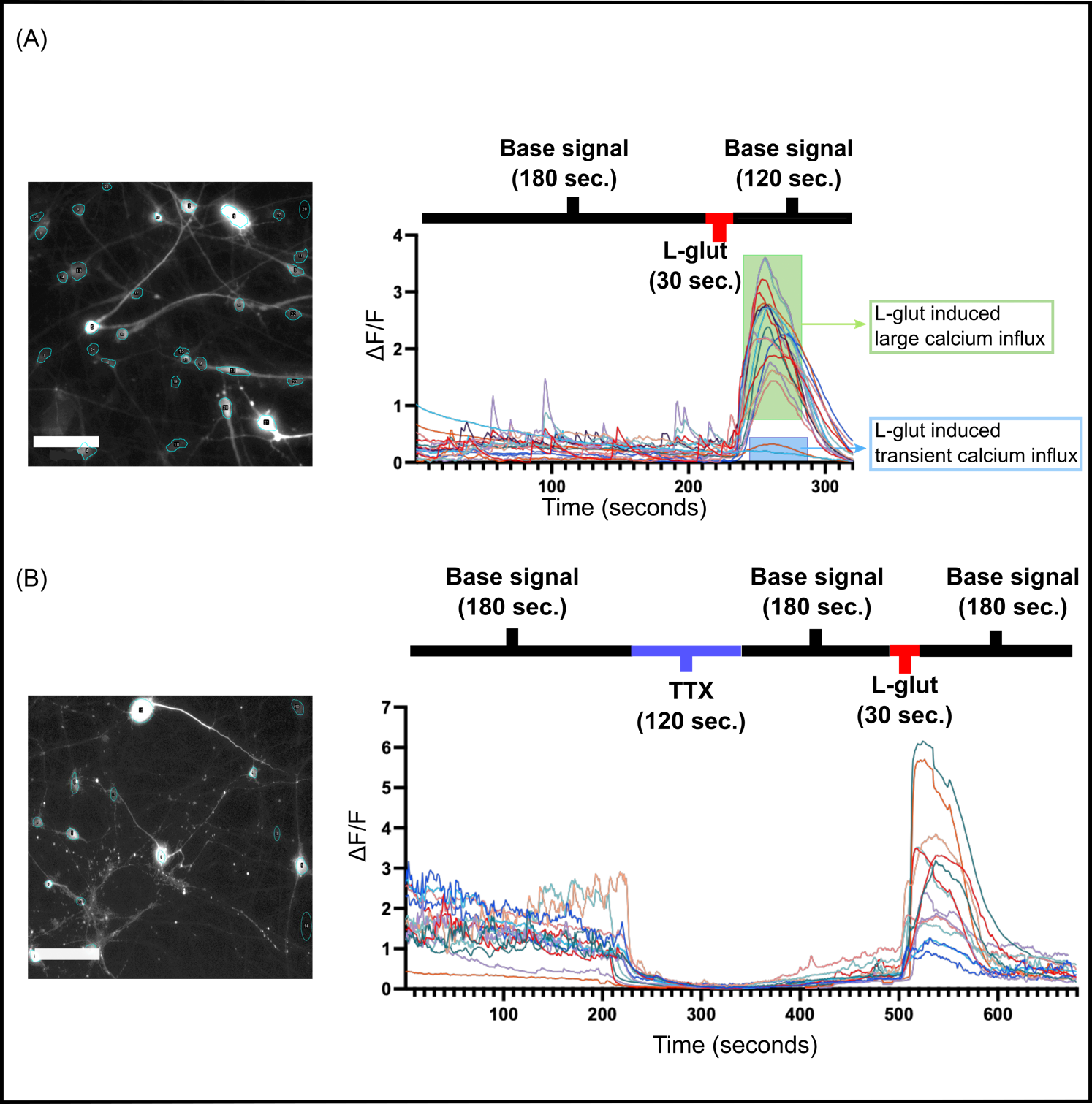


(A) The left image shows the recording area of the neural culture and the ROIs marked around the cell body. The left graph shows representative traces of live-cell Ca^2+^ imaging on week 3. After 180 seconds, cells were stimulated with 30 μM / 30 seconds L-glut, leading to a large influx of Ca^2+^ in neurons. This response allowed distinguishing neurons from non-neuronal cells (indicated by the green box). However, some non-neuronal cells also showed only a transient increase or no response (indicated by the blue box) in fluorescent upon glutamate application. (B) To further confirm the relationship between neuronal activity and Ca^2+^ activity, some neural cultures were treated with 1 µM TTX for 120 seconds to block the voltage-gated sodium channels and then washed with basal medium before being stimulated with glutamate application at 30 µM / 30 seconds. The application of TTX significantly inhibited the generation of neuronal spontaneous Ca^2+^ events, suggesting that the Ca^2+^ spontaneous events are a secondary effect arising from voltage-gated sodium channel-mediated neuronal activity (scale bars = 50 μm).
